# Phenome-wide Analysis of Diseases in Relation to Objectively Measured Sleep Traits and Comparison with Subjective Sleep Traits in 88,461 Adults

**DOI:** 10.34133/hds.0161

**Published:** 2025-06-03

**Authors:** Yimeng Wang, Qiaorui Wen, Siwen Luo, Lijuan Tang, Siyan Zhan, Jia Cao, Shengfeng Wang, Qing Chen

**Affiliations:** ^1^ Key Lab of Medical Protection for Electromagnetic Radiation, Ministry of Education of China, Institute of Toxicology, College of Preventive Medicine, Third Military Medical University (Army Medical University), Chongqing 400038, China.; ^2^ Department of Epidemiology and Biostatistics, School of Public Health, Peking University Health Science Center, Beijing 100191, China.; ^3^ Key Laboratory of Epidemiology of Major Diseases (Peking University), Ministry of Education, Beijing 100191, China.; ^4^ Department of Breast and Thyroid Surgery, Daping Hospital, Army Medical University, Chongqing 400010, China.

## Abstract

**Background:** Sleep traits have been suggested to correlate with various diseases, but most evidence is based on subjective sleep measurement. We investigated the associations of accelerometer-derived objective sleep traits with diseases throughout physiological systems to ascertain whether the disease spectrum related to objective sleep traits differs from that related to subjective sleep traits. **Methods:** In 88,461 UK Biobank (UKB) adults wearing accelerometers, multiple dimensions of sleep were objectively derived: (a) nocturnal sleep duration and onset timing, (b) sleep rhythm (relative amplitude and interdaily stability), and (c) sleep fragmentation (sleep efficiency and waking numbers). Associations with International Classification of Diseases, 10th Revision–decoded diseases during follow-up were estimated using the Cox model, and the results were compared with those of a published literature search of subjectively measured sleep traits and diseases. National Health and Nutrition Examination Survey (NHANES) data were used to validate the newly identified associations unreported by previous studies. For the meta-analysis-reported associations (with subjective sleep traits) that were negative (with objective sleep traits) in our study, reanalysis was done in UKB with subjective sleep traits, stratified by objective measurements. **Results:** During the average 6.8-year follow-up, 172 diseases were associated with sleep traits. Among them, 42 showed at least doubled disease risk, including age-related physical debility (lowest versus highest quartile of relative amplitude, hazard ratio [HR] = 3.36, 95% confidence interval [CI]: 2.25, 5.02), gangrene (lowest versus highest quartile of interdaily stability, HR = 2.61, 95% CI: 1.41, 4.83), and fibrosis and cirrhosis of the liver (sleep onset timing ≥0030 versus 2300 to 2330, HR = 2.57, 95% CI: 1.42, 4.67). A total of 92 diseases had >20% burden attributable to sleep, such as Parkinson’s disease (37.05%, 95% CI: 21.02%, 49.83%), type 2 diabetes (36.12%, 95% CI: 29.00%, 42.52%), and acute kidney failure (21.85%, 95% CI: 13.47%, 29.42%). Notably, 83 (48.3%) disease associations were sleep rhythm specific, distinct from existing subjective-measure literature that focused on sleep duration. Reanalysis in UKB showed a contamination of objectively short sleepers in self-report long sleepers, which induced false-positive associations in subjective meta-analyses, including for ischemic heart disease and depressive disorder. Newly identified associations of sleep rhythm with 4 diseases including chronic obstructive pulmonary disease and diabetes were successfully replicated in NHANES. A mediation analysis showed that inflammatory factors including leukocytes, eosinophils, and C-reactive protein contributed significantly to all these newly identified sleep–disease associations. **Conclusions:** Objective sleep traits showed a disease spectrum similar to but not identical to that of subjective sleep traits. Objective measurement can be a useful complement to sleep–disease studies as it may help overcome false-positive associations caused by misclassification bias of some subjective measurement such as sleep duration. Comprehensive control of multiple sleep traits may be important for health as substantial disease burden was attributed to different sleep traits.

## Introduction

Sleep is a fundamental life requirement and is critical for multiple biological functions, including brain waste clearance [1], inflammatory cytokine release [2], and nutrient metabolism [3]. With the widespread use of artificial lighting and the “24/7” lifestyle, the human sleep experience has significantly changed from that of our pre-industrial revolution ancestors [4,5]. Not only sleep duration but also the timing, regularity (rhythmic robustness), and continuity (fragmentation) of sleep have also been profoundly affected [6]. Whether the human physiological systems that evolved over deep history could adapt to the current sleep pattern is a question that deserves specific attention for its potential impact on human health [7].

Indeed, accumulating evidence suggests that sleep traits might be associated with a number of diseases affecting diverse systems, such as cardiovascular diseases, metabolic disorders, and mental diseases [8,9]. However, in most publications to date, sleep has been measured by subjective recall of the participants, which has been found to bear systematic error in some aspects and may lead to misunderstanding of sleep’s health impact [10]. Furthermore, the available literature has mainly focused on certain sleep traits, namely, sleep duration, while other traits such as sleep timing and sleep rhythm have less often been analyzed, despite animal studies and some pilot studies in human beings having suggested that disruption of other sleep traits also exerts substantial effects on physiological homeostasis [11,12].

The development of objective sleep measurement technology such as accelerometry has provided an alternative choice, which is capable of measuring the indicators of different sleep traits at one time and has shown higher accuracy in the measurement of some sleep traits [13]. However, until now, there has been no study comparing objective sleep measurement to subjective sleep measurement in terms of their association with diseases throughout different systems.

**Fig. 1.**
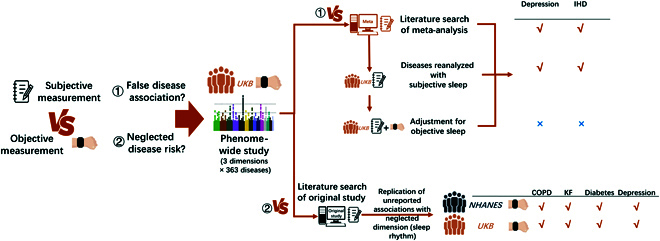
Flow chart of the study. Firstly, in 88,461 UK Biobank (UKB) adults wearing accelerometers, a prospective phenome-wide analysis was conducted of 363 International Classification of Diseases, 10th Revision–decoded diseases and 3 dimensions of sleep traits: (a) nocturnal sleep duration and onset timing, (b) sleep rhythm (relative amplitude and interdaily stability), and (c) sleep fragmentation (sleep efficiency and waking numbers). The results were compared with those of a literature search of subjectively measured sleep traits and diseases, and 2 separate investigations were carried out. (a) For the meta-analysis-reported associations that were negative in our study, reanalysis was done in the UKB with subjective sleep traits, stratified by objective measurements. Two diseases (depression and ischemic heart disease [IHD]) were found to have false-positive associations with subjective sleep duration in the UKB, whose associations were eliminated when adjusted by objective sleep duration. (b) Newly identified associations unreported by previous studies (of chronic obstructive pulmonary disease [COPD], kidney failure [KF], diabetes, and depression with sleep rhythm) were validated in the National Health and Nutrition Examination Survey (NHANES) dataset. All 4 associations were successfully replicated. Depression indicates depressive episode and recurrent depressive disorder.

To begin addressing the abovementioned gaps, we conducted a comprehensive analysis of the relationships between various adverse outcomes and multiple objectively measured sleep traits based on the UK Biobank (UKB) cohort and compared the findings with those in previous publications concerning diseases and subjective sleep traits. The goal of this analysis was to determine whether the disease spectrum related to objective sleep traits differ from that related to subjective sleep traits and to quantitatively estimate the disease contribution of different objective sleep traits (Fig. 1). Meanwhile, the National Health and Nutrition Examination Survey (NHANES) was used to replicate the previously unreported trait–disease associations found in our objective trait–disease analyses in the UKB. This research may provide new insights for future sleep-related studies in terms of the selection of sleep measurement methods.

## Methods

### Phenome-wide analysis of objective sleep traits and diseases

#### Study population

Data were sourced from the UKB study. About half a million residents were enrolled in 2006 to 2010 from 22 study centers across the United Kingdom, as described in detail elsewhere [14,15]. Accelerometer data were drawn from a subsample of 103,666 participants who wore a wrist-worn AX3 triaxial accelerometer (Axivity, Newcastle upon Tyne, UK) for 7 d during 2013 to 2015. We excluded unqualified accelerometry data, after which 88,461 participants remained for the main analysis (Fig. S1). Details on the accelerometer device used are shown in Supplementary Methods.

#### Measurement of sleep traits

As suggested by previous studies, 3 dimensions including 6 sleep traits were derived to represent different aspects of sleep measures: (a) nocturnal sleep duration and sleep onset timing [16], (b) sleep rhythm (including relative amplitude and interdaily stability) [17], and (c) sleep fragmentation (including sleep efficiency and nocturnal waking number, which represents the number of awakenings during the sleep period time window) [18]. All sleep traits were derived by processing raw accelerometer data using GGIR (version 2.8.2) [16,19].

#### Measurement of covariates

Sociodemographic information (sex, ethnicity, education qualification attainment, total household income, and employment status), lifestyle behaviors (physical activity; smoking status; alcohol drinking; consumption of tea, coffee, and sugar products; and intakes of vegetables, fruits, fish, red meat, and processed meat), medication at baseline, and illness of father, mother, and siblings were all self-reported. Age was calculated as the intervals between the dates of birth and of accelerometer measurements. The Townsend deprivation index was derived from the postcode of residence. Standing height (cm) and weight (kg) were measured at the assessment center and used to calculate the body mass index (BMI).

#### Follow-up of diseases

Diagnosis of outcome was ascertained through linkage to the National Health Service and Cancer Registry for any episodes of hospitalization or to the National Death Index for date and causes of death. Details of the linkage procedure are available online (https://digital.nhs.uk/). All reported disease events were coded following the International Classification of Diseases, 10th Revision or 9th Revision (ICD-10 or ICD-9). Events coded by ICD-9 were converted to ICD-10 (https://www.cms.gov/). Follow-up began with the date when participants completed accelerometer-based measurements. Participants with specific diseases before that date were excluded from related analyses. Participants were censored on death or the most recent hospitalization (2021 September 30 for England and Wales and 2021 September 24 for Scotland), whichever came first.

All disease events coded according to the first 3 characters of ICD-10 were selected and combined based on knowledge about the diseases when appropriate. Certain irrelevant ICD-10 chapters were excluded, including those on perinatal diseases, congenital malformations, deformations, and chromosomal abnormalities. Within each chapter, outcomes with <100 events (<80 for sex-specific analyses) were combined. These outcomes were not analyzed in the detailed investigation of sleep traits and individual diseases. The detailed list of ICD-10 codes analyzed is given in Table S1.

#### Statistical analysis

The Cox proportional hazard model was applied to estimate hazard ratios (HRs) and 95% confidence intervals (CIs) for the association of sleep traits and diseases, with stratification for age (in 10-year intervals) and sex, along with adjustment for ethnicity; education qualification attainment; total household income; employment status; smoking status; alcohol drinking; tea consumption; coffee consumption; sugar consumption; intakes of vegetables, fruits, fish, red meat, and processed meat; physical activity (metabolic equivalent hours per day); medication for cholesterol, blood pressure, or diabetes at baseline; usage of sleep medications, antidepressants, antipsychotics, or anxiolytics; BMI; and family history of related diseases [20]. To screen disease chapters associated with sleep traits while avoiding type I errors, the relatively conservative Bonferroni correction method was employed for association analysis. The significance threshold was defined as 0.05 divided by the number of sleep trait categories (excluding the reference categories) and the number disease chapters. For those disease chapters that showed significant association with at least one sleep trait after Bonferroni’s correction, the associations of each sleep trait with individual diseases within the chapter were further examined where the significance threshold was set as 0.05 after false discovery rate (FDR) correction method (Benjamini–Hochberg procedure).

Assuming that each observed association is causal, we further calculated the population-attributable fraction (PAF) for each sleep trait on the associated diseases [21]. We coded sleep traits as binary variables and compared participants with the least risky level of sleep trait against all others, as suggested by Newson [22].

Stratification analysis was additionally performed for the identified trait–disease associations based on age (<65 versus ≥65 years), biological sex (women versus men), education (college and above versus less than college), and chronotype (morningness versus eveningness). Four sensitivity analyses were conducted separately by excluding (a) those who reported jobs involving shift work or night shift work [18]; (b) those who reported using sleep medications, antidepressants, antipsychotics, or anxiolytics at baseline [18]; (c) those whose accelerometer-wearing period included a daylight-saving time change or who woke up in the afternoon on an average day [23,24]; and (d) those who were lost or developed diseases within the first 2 years of follow-up. Post hoc analysis was conducted by calculating the E-value ($E-\text{value}=\text{RR}+\sqrt{\text{RR}\times \text{RR}-1}$, where RR represents the observed relative risk) [25], which quantitatively estimated how strong the effect size of an unmeasured confounder need to be, so that the main association that we analyzed would be explained away by the unmeasured or residual confounder [26]. The larger the E-value, the less likely that the study results were explained by confounding. For factors that showed inverse associations with disease outcomes, E-values were calculated using 1/RR instead of RR. All statistical analyses were performed using R v4.2.2 and Stata 16.0 (StataCorp, TX, USA).

### Comparison with studies of subjective sleep measurement

#### Target of comparison: Literature search of subjective sleep traits and diseases

A literature search was conducted to retrieve research articles or meta-analyses that investigated the association of diseases with subjectively measured sleep traits, referring to any of the 3 sleep dimensions analyzed in our study. A systematic search was conducted using PubMed and Web of Science for articles published before March 2024. Search terms included “sleep AND X”, “sleep duration AND X”, “sleep onset AND X”, “circadian rhythm AND X”, “social jetlag AND X” (social jetlag being a term commonly used to indicate sleep rhythm disruption), “sleep efficiency AND X”, and “waking time AND X”, wherein X stands for a specific disease, like cystitis. The a priori inclusion criteria were defined as any study that (a) exclusively employed subjective measurement methods, such as questionnaires or interviews, to assess the sleep traits of participants; (b) focused on the disease of interest as the primary outcome; (c) calculated effect sizes and statistical significance; and (d) was written in English. There are another 106 diseases named abstractly, such as “other respiratory disorders”, and 57 diseases belonging to “symptoms, signs and abnormal clinical and laboratory findings, not elsewhere classified” (Chapter XVIII), which were not to be included. Original research and meta-analyses were separately summarized. Since the aim of the literature search was to identify previously overlooked associations between sleep traits and diseases, those diseases showing no trait–disease association in our study were omitted. In comparison to the literature search, the inconsistent trait–disease relationships in our results were further reanalyzed or replicated with an independent population, as detailed in the following sections.

#### Identification of false disease associations: Reanalysis with subjective sleep traits in UKB participants

For sleep trait–disease pairs found to have positive association in subjective meta-analyses but not our objective analysis, we performed a reanalysis based on subjective sleep traits in the UKB. The subjective sleep traits were collected by questionnaire, as described in detail elsewhere [27]. When multiple meta-analyses were available for a trait–disease pair, only the consistent results were reexamined. If the results of reanalysis aligned with the meta-analysis rather than the objective results, the reanalysis results were further stratified by the objective sleep trait so as to investigate whether the association with subjective sleep traits was caused by misclassification of objective sleep traits. Similarly, negative associations in meta-analyses but not in our analysis were likewise reanalyzed. Finally, correlations of subjective and objective sleep traits were analyzed.

#### Validation of neglected disease associations: Independent replication analysis

We plotted the disease types associated with each objective sleep dimension and compared these patterns with findings from subjective sleep literature to determine whether any dimensions were overlooked in subjective studies. For such dimensions, the significant disease associations identified based on objective sleep traits in the UKB but not reported by previous subjective studies were replicated in the NHANES dataset. During the 2011 to 2012 and 2013 to 2014 cycles of NHANES, objective accelerometer records were collected from the participants (ActiGraph model GT3X+, Pensacola, USA) [22]. We selected a representative sample of 8,514 adults aged ≥20 years from the NHANES dataset using a complex, stratified, multistage sampling design and the same criteria as applied in the UKB study. Sleep traits were estimated using the monitor-independent movement summary method, which was run with the ActCR package in R. Covariate selection also followed the analysis in the UKB population where applicable, with covariates including age, sex, ethnicity, education qualification attainment, ratio of family income to poverty, employment status, smoking status, alcohol drinking, physical activity, and BMI [28]. NHANES generally collected disease occurrence information by questionnaire. The list of diseases and the questionnaire used can be found online (https://www.cdc.gov/nchs/nhanes). According to NHANES analytic guidelines, sample weights, strata, and primary sampling units were used to account for the complex survey design. Statistical analysis was generally the same as in the UKB analysis, except that trait–disease relationships were analyzed using weighted multivariable-adjusted logistic regression instead of a Cox model. Finally, mediation analysis was used to explore the potential pathological mechanisms of newly identified associations, specifically whether immune factors, including leukocytes, C-reactive protein (CRP), and eosinophils mediate the associations in the UKB. This mediation analysis method was developed based on the Cox model and under a counterfactual framework [29,30].

### Role of the funding source

The funders had no role in the study design, data collection, data analysis, data interpretation, or writing of the report.

## Results

### General characteristics and sleep traits of the participants

The 88,461 included participants were generally comparable to those excluded, except for slight differences in age and employment status (Table S2). The included participants were on average 61.97 years (standard deviation, 7.83 years) old, and 43.42% were males. Figure S2 illustrates the distribution of the sleep traits (Fig. S2A) and their pairwise correlations (Fig. S2B).

### Phenome-wide association analysis of diseases related to sleep traits

**Fig. 2.**
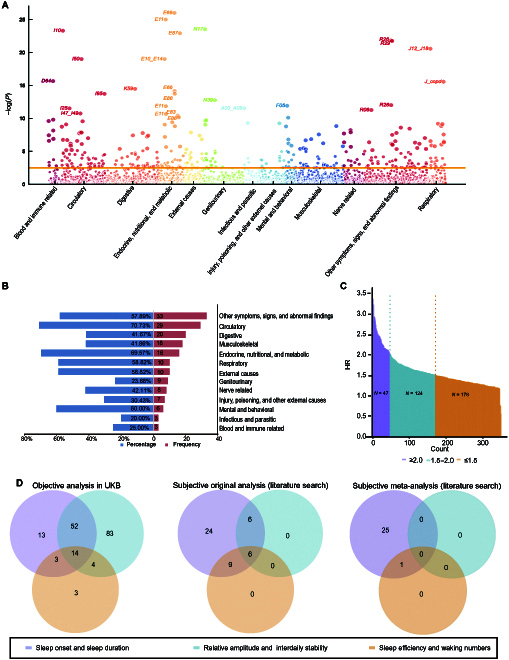
Phenome-wide association analysis of 363 candidate diseases related to 6 traits of sleep. (A) Adjusted *P* values for risk of disease incidence across the 13 ICD-10 chapters (total 363 candidate diseases). The false discovery rate (FDR) threshold for multiple comparisons is shown as a solid horizontal line. (B) Percentage and frequency of diseases correlating with at least one sleep pattern trait by chapter. (C) Distribution of adjusted hazard ratios (HRs) for sleep-associated diseases. Diseases are ordered based on the adjusted HR. (D) Summary of diseases and sleep dimensions by objective sleep measurement in our study (left), subjective sleep measurement in previous original research (middle), and subjective sleep measurement in meta-analyses (right). Diseases related to multiple sleep traits in the same dimension were counted just once.

During an average 6.80 ± 0.87 years of follow-up, 113,146 person-diseases were newly diagnosed (Table S3). After Bonferroni correction, 13 of the 17 disease chapters showed significant association with at least one sleep trait; examples include mental and behavioral disorders, circulatory system diseases, and digestive system diseases (Fig. S3 and Table S4). Four chapters, including neoplasms (Chapter II; *P* = 0.00096), were excluded from further analysis as they did not meet the adjusted significance threshold (*α* = 0.00049). Collectively, the 13 chapters comprised a sum of 363 candidate diseases. Multivariate analysis showed that 172 (47.38%) of those diseases were associated with at least one sleep trait, for a cumulative 349 trait–disease associations (Fig. 2A and Table S5). The top 5 chapters with the most trait-related diseases were abnormal clinical and laboratory findings (Chapter XV, *n* = 33), circulatory diseases (Chapter IX, *n* = 29), digestive diseases (Chapter XI, *n* = 20), musculoskeletal and connective diseases (Chapter XIII, *n* = 18), and endocrine, nutritional, and metabolic diseases (Chapter IV, *n* = 16). The respiratory system (Chapter X) and genitourinary system (Chapter XIV), which were seldom reported in sleep studies, also respectively had 10 and 9 disease–trait associations (Fig. 2B). A total of 42 diseases exhibited over 2-fold risk associated with a certain sleep trait, such as Parkinson disease (G20, lowest versus highest quartile of relative amplitude, HR = 2.80, 95% CI: 1.96, 4.02), age-related physical debility (R54, lowest versus highest quartile of relative amplitude, HR = 3.36, 95% CI: 2.25, 5.02), gangrene (R02, lowest versus highest quartile of interdaily stability, HR = 2.61, 95% CI: 1.41, 4.83), and fibrosis and cirrhosis of liver (K74, sleep onset timing ≥0030 versus 2300 to 2330, HR = 2.57, 95% CI: 1.42, 4.67), accounting for 11.57% of all diseases analyzed and 24.42% of those found to be associated with sleep; meanwhile, 122 diseases (33.61% of all diseases and 70.93% of sleep-associated diseases) had at least 1.5-fold risk (Fig. 2C), including type 2 diabetes mellitus (E11, lowest versus highest quartile of interdaily stability, HR = 1.60, 95% CI: 1.40, 1.82), respiratory failure (J96, lowest versus highest quartile of sleep efficiency, HR = 1.79, 95% CI: 1.42, 2.26), fracture of rib(s), the sternum, and the thoracic spine (S22, sleep duration <6 h versus 7 to 8 h, HR = 1.60, 95% CI: 1.22, 2.11), and urinary incontinence (R32, sleep duration <6 h versus 7 to 8 h, HR = 1.60, 95% CI: 1.25, 2.04).

PAF was estimated for each sleep trait and its associated diseases (Table S6). Among the 172 diseases found to be associated with sleep, up to 52.29% of the risk of a single disease could be attributed to a sleep trait, 92 (53.49%) diseases had over 20% risk attributable to at least one sleep trait, and 44 (25.58%) diseases had attributable risk over 30% (Fig. S4). Some common diseases showed considerable attributable risk, such as Parkinson disease (G20; 37.05% by interdaily stability), pulmonary heart diseases (I27; 49.91% by relative amplitude), type 2 diabetes mellitus (E11; 22.39% by interdaily stability), obesity (E66; 31.63% by relative amplitude), thyrotoxicosis (E05; 30.45% by sleep onset), and urinary incontinence (R32; 24.42% by sleep efficiency).

Stratified analyses were performed for sex, age, education, and chronotype. The results were similar between stratifying variables, and no significant interaction was observed (in Tables S7 to S10). To exclude the potential bias introduced by shift work, sleep-related medications, or daylight-saving time, sensitivity analyses were performed by excluding the respectively exposed adults; the relationships of sleep traits and diseases were generally unchanged (Table S11). Participants who were lost or developed diseases within the first 2 years of follow-up were also excluded in a separate round, and again, the results were alike (Table S11). In general, the results showed that the trait–disease relationships were robust compared to the main analysis across all these sensitivity analysis. Parkinson’s disease (G20, lowest versus highest quartile of relative amplitude, HR = 3.00, 95% CI: 2.07, 4.35 in model 1; HR = 3.50, 95% CI: 2.22, 5.53 in model 2; HR = 2.84, 95% CI: 1.96, 4.13 in model 3; HR = 2.68, 95% CI: 1.82, 3.94 in model 4) and age-related physical debility (R54, lowest versus highest quartile of relative amplitude, HR = 3.31, 95% CI: 2.20, 4.98 in model 1; HR = 4.65, 95% CI: 2.78, 7.76 in model 2; HR = 3.29, 95% CI: 2.17, 4.99 in model 3; HR = 3.26, 95% CI: 2.18, 4.88 in model 4) can be 2 examples. The E-values for FDR-adjusted statistically significant associations between sleep traits and diseases are in Table S12; these values indicate what strength of association an unmeasured confounder would need to have with exposures and morbidity for our estimates to fit the null.

In total, the 172 diseases showed 259 associations with sleep traits, among which the dimensions of sleep duration and onset timing accounted for 82 (31.7%), while sleep rhythm and sleep quality accounted for the rest (Fig. 2D, left). This is distinct from the literature search of subjective sleep traits and disease risk (original research studies), which showed that sleep duration or sleep timing were associated with 45 diseases, accounting for 57.7% of all the interested trait–disease pairs, while sleep rhythm was associated with 12 diseases, accounting for only 15.4% (Fig. 2D, middle, and Table S13). Notably, the diseases tended to not be affected by multiple dimensions of sleep, with 99 (57.6%) diseases showing association with only a single sleep dimension.

### Identification of false disease associations

#### Comparison to published meta-analyses based on subjective sleep

**Fig. 3.**
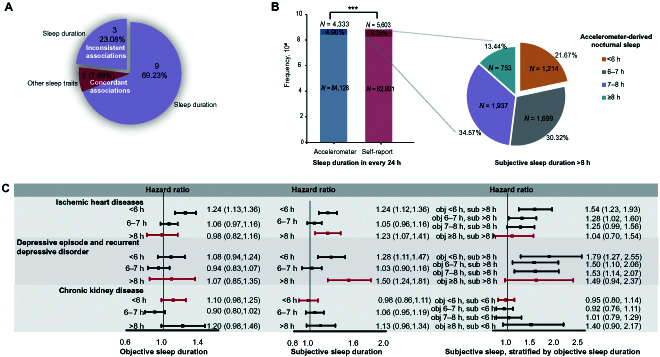
Comparison of objective sleep measurement to self-reported sleep. (A) Published meta-analysis of subjective sleep traits and diseases. Purple pieces represent the associations between disease and sleep duration. The red piece represents the association between disease and other sleep traits. The separate piece (23.08%) represents those sleep–disease associations that were inconsistent between meta-analyses and our study with objective sleep traits. (B) Comparison of objectively long sleep duration (≥9 h) to self-reported long sleep duration. The bar plot illustrates the proportion of long sleepers by objective accelerometer and subjective recall, compared by chi-square test (*** indicates *P* < 0.01). The pie plot illustrates the composition of subjectively long sleepers, within which objectively short sleepers (<6 h) accounted for 21.67%. (C) Reanalysis of the subjective–objective-inconsistent diseases in the UKB based on subjective recall of sleep traits. Three trait–disease associations were found to be negative in our objective analysis but positive in previous meta-analyses, including long sleep duration with ischemic heart disease, long sleep duration with depressive episode and recurrent depressive disorder, and short sleep duration with chronic kidney disease. Reanalysis of these diseases based on subjective sleep traits in the UKB recapitulated the 2 associations with subjectively long sleep duration ((C) middle; error bar in red) rather than the non-association of objective measurement ((C) left; error bar in red). A further analysis examined the association of these diseases with subjectively long sleep (short sleep for chronic kidney disease), stratified by objective sleep duration ((C) right; error bar in red). For the upper 2 diseases (ischemic heart disease; depressive episode and recurrent depressive disorder), subjectively long sleepers were divided into 4 groups based on objective nocturnal sleep duration (<6, 6 to 7, 7 to 8, and ≥8 h), and their disease risk was separately compared to those who reported a 7- to 8-h subjective sleep duration. For chronic kidney disease (below), the reanalysis was done in subjectively short sleepers, although subjectively short sleep was not found to be associated with the disease in the UKB, as shown in (C) (middle). obj, objective; sub, subjective.

A meta-analysis-specific literature search for reports associating subjective sleep traits with disease risks was performed. A total of 26 trait–disease pairs were analyzed by previous meta-analyses (Table S14 and Fig. 2D, right). The involved diseases were generally common diseases such as dementia, chronic kidney disease, osteoporosis, essential (primary) hypertension, type 2 diabetes, and stroke. For 9 sleep–disease relationships, such as short sleep duration and osteoporosis, there were more than one meta-analysis, which investigated the same sleep trait but showed inconsistent conclusions; i.e., negative and positive associations were reported in different meta-analyses. Sleep duration was referred to by all included meta-analyses, while other sleep traits were analyzed in only one of them. As shown in Table S14, when compared to our results, most diseases showed concordant positive association with subjective sleep traits, such as short sleep duration in association with dementia, essential (primary) hypertension, heart failure, overweight and obesity, and type 2 diabetes. However, 3 diseases were associated with subjective sleep duration but not its objective measurement (Fig. 3A and C, left). Among those, 2 diseases (ischemic heart diseases and depressive episode and recurrent depressive disorder) were associated with long sleep and the third (chronic kidney disease) with short sleep. Meanwhile, no negative trait–disease relationship in our study was reported to be significantly positive by previous meta-analyses (Table S14).

#### Reanalysis of the negative trait–disease associations that were positive in previous meta-analyses

We reanalyzed the 3 inconsistently associated diseases in relation to subjective recall of sleep duration in the UKB participants. To ensure the comparability of the results, we restricted analysis in the participants who had qualified accelerometer data. The results recapitulated the significant associations of subjectively long sleep duration with both ischemic heart diseases and depressive episode and recurrent depressive disorder (Fig. 3C, middle), which was in agreement with the meta-analysis results.

Then, we investigated what may induce the different results between subjective and objective sleep durations with respect to the 3 diseases. Pearson analysis showed that the 2 measurements showed a mild correlation only (*r* = 0.201, *P* < 0.00001). The proportion of long sleepers (>8 h) is 1.29-fold more by self-report compared to that by objective measurement (Fig. 3B). When the 5,603 self-reported long sleepers were further investigated, as many as 21.67% of them obtained no more than 6 h of objective sleep, which should be defined as short sleep (Fig. 3B). Since short sleep duration had been widely perceived as harmful, further analysis was conducted to determine whether the association between subjectively long sleep and disease risk remained when stratified by objective sleep duration. The results showed that the increased risk of ischemic heart diseases in subjectively long sleepers existed only in this subset of objectively short sleepers (HR = 1.54, 95% CI: 1.23, 1.93) (Fig. 3C, right), while for the both subjectively and objectively long sleepers, the effect size (HR) was 1.04 (95% CI: 0.70, 1.54). When the subset of objectively short sleepers was excluded, the increased risk in subjectively long sleepers disappeared. A similar result was obtained in the analysis of depressive episode and recurrent depressive disorder, where objectively short sleepers contributed the highest effect size (HR = 1.79, 95% CI: 1.27, 2.55), while the objectively long sleepers did not achieve statistical significance (HR = 1.49, 95% CI: 0.94, 2.37) (Fig. 3C, right). As a recent publication of UKB adults reported that subjectively long sleep duration is associated with multiple cardiovascular diseases, including but not limited to aforementioned ischemic heart diseases [27], we further extended the reanalysis to stroke and cardiovascular disease (that is, ischemic heart diseases and stroke). Again, the increased risk of stroke and cardiovascular diseases in the self-reported long sleepers disappeared when the subset of objectively short sleepers was removed (Fig. S5). The effect sizes (HR) on stroke and cardiovascular diseases were 1.06 (95% CI: 0.57, 2.00) and 1.09 (95% CI: 0.78, 1.54) for objectively and subjectively long sleepers, respectively. The association of subjectively short sleep with chronic kidney disease was not found in the UKB (Fig. 3C), either before or after stratification by objective measurement.

### Validation of neglected disease associations

#### Comparison to literature search of original studies using subjective sleep

Comparison with the existing literature on subjective sleep traits and disease risk showed that among the 172 sleep-related diseases identified in our study, 90 presented associations with sleep trait dimensions that had not been reported before (Table S13), including acute kidney failure (N17) and chronic obstructive pulmonary disease (COPD). Sleep rhythm (relative amplitude and interdaily stability) contributed to most (61) of the newly discovered associations, while sleep duration/onset and sleep quality contributed to 20 and 9 diseases, respectively (Fig. S6).

#### Replications in an independent population

**Fig. 4.**
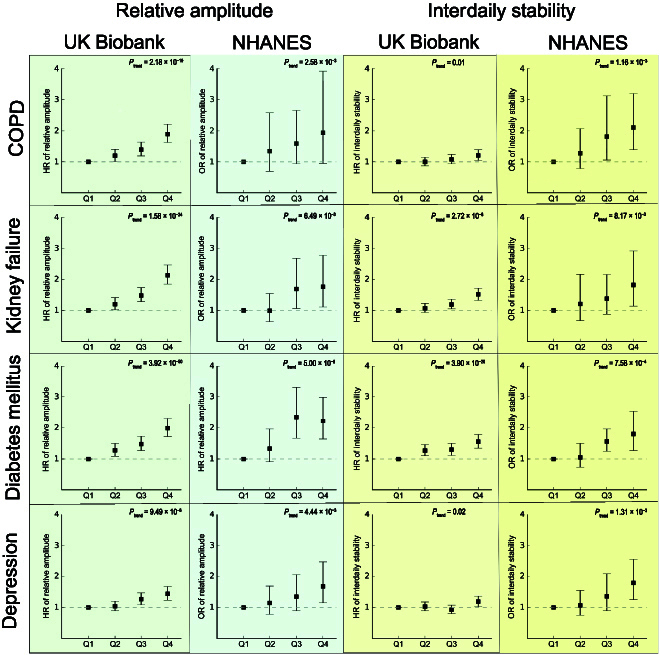
Replication of the newly discovered associations with sleep rhythm in the NHANES study. HRs and 95% confidence intervals (CIs) for the identified associations of relative amplitude and interdaily stability were calculated in the UKB sample, with adjustment for age; sex; race; education; total household income; employment status; smoking status; alcohol drinking; tea consumption; coffee consumption; sugar consumption; intakes of vegetables, fruits, fish, red meat, and processed meat; physical activity; medication for cholesterol, blood pressure, or diabetes at baseline; usage of sleep medications; BMI; and family history of chronic bronchitis/emphysema in COPD. As a replication analysis, the odds ratios and 95% CI for these associations were calculated in the NHANES participants, with adjustment for age, sex, race, education, ratio of family income to poverty, employment status, smoking status, alcohol drinking, physical activity, and BMI. The NHANES data were weighted by sample weights, strata, and primary sampling units following the suggestion of the data provider. The *P* values for linear trends were calculated by taking the sleep trait quartiles as continuous variables. OR, odds ratio.

Of the diseases that were newly found to be associated with sleep rhythm, 4 were also measured in NHANES: acute kidney failure, COPD, diabetes mellitus, and depressive episode and recurrent depressive disorder. Accordingly, we validated their associations with sleep rhythm in the NHANES population (Fig. 4). Detailed descriptions of the NHANES population are presented in Table S15. Relative to the UKB population, the NHANES population are on average younger and have a higher BMI. In the UKB population, acute kidney failure was associated with relative amplitude and interdaily stability in the dose–response pattern (*P*_trend_ = 1.58 × 10^−24^ and 2.72 × 10^−8^, respectively). Likewise, in the NHANES population, the third and fourth quartiles of relative amplitude were associated with 1.69-fold (95% CI: 1.06, 2.69) and 1.76-fold (95% CI: 1.11, 2.78) greater kidney failure risk (*P*_trend_ = 0.017), respectively, and the fourth quartile of interdaily stability with 1.82-fold (95% CI: 1.14, 2.82) greater kidney failure risk (*P*_trend_ = 8.17 × 10^−3^). The relationship between relative amplitude and COPD detected in the UKB was similarly seen in NHANES (*P*_trend_ = 0.026) with the third and fourth quartiles of relative amplitude being associated with 1.59-fold (95% CI: 0.95, 2.65) and 1.94-fold (95% CI: 0.96, 3.93) increased COPD risk, respectively. As for diabetes mellitus, relative amplitude and interdaily stability presented dose–response patterns (*P*_trend_ = 3.92 × 10^−50^ and 3.90 × 10^−20^, respectively) in the UKB population. Likewise, in the NHANES population, the third and fourth quartiles of relative amplitude and interdaily stability were associated with diabetes mellitus (*P*_trend_ = 5.00 × 10^−8^ and 7.58 × 10^−4^, respectively). Finally, a consistent relationship of relative amplitude with depressive episode and recurrent depressive disorder was also observed in both populations (*P*_trend_ = 9.49 × 10^−6^ and 4.44 × 10^−3^).

The unweighted results from the NHANES population are similar to the weighted results (Fig. S7).

#### Mediation analysis

To explore potential mechanisms for the newly identified associations of diseases with sleep rhythm, we examined inflammatory factors as mediators (Table S16), including leukocytes, eosinophils, and CRP. The results showed that CRP contributed a statistically significant mediation effect to all the 4 diseases’ associations with sleep rhythm (*P* < 0.001, respectively), and CRP’s contribution ranged from 1.5% to 10.5%. Leukocytes and eosinophils generally contributed especially to acute kidney failure and COPD but not diabetes mellitus or depressive episode and recurrent depressive disorder. All 3 inflammatory factors contributed a mediation effect for COPD and acute kidney failure. For example, to acute kidney failure, the 3 inflammatory factors CRP, eosinophils, and leukocytes respectively contributed 10.5%, 8.2%, and 1.9% of the effect.

## Discussion

Based on a dataset of 88,461 adults followed up for averagely 6.8 years, the present study showed that objectively measured sleep traits are associated with 172 diseases affecting diverse systems of the human body and that these associations account for considerable attributable disease burden, up to 52%. Although the associations were generally consistent with those in previous studies based on subjectively measured sleep traits, notable differences emerged, especially in the analysis of long sleep duration, where our objectively measured sleep duration barely showed disease risk. Among the various associations, 57.6% of diseases correlated with only one dimension of sleep traits, and the most concerned sleep trait (sleep duration) by subjectively measured studies did not contribute to the most diseases. On the other hand, the analysis of objective sleep traits identified a number of disease associations not ever reported in previous sleep studies, including the associations of sleep rhythm with COPD and acute kidney failure. These associations were replicated in the independent NHANES population (Fig. 1). These results provide important insights into the landscape of sleep’s health impact on the human body and suggest that the objective sleep measurement technique can be a useful complement to sleep–disease relationship studies.

The similarity and difference between subjective and objective sleep measurement is a fundamental methodology issue for all sleep-related studies. In some cases, the relationships of certain diseases with both subjective and objective sleep traits have been analyzed and compared [31–37]. For example, in 7,959 participants of the Whitehall II study, both subjectively and objectively short sleep durations were found to be associated with higher incidence of dementia [33]. Meanwhile, for some other diseases, the objective and subjective measurements did not achieve agreement in association with diseases [33,34,36]. With the existing publications, it is difficult to determine whether similarity or difference is dominant across associations with various diseases, since there has been no study to compare objective and subjective measurements in light of diseases throughout the human body, according to the knowledge of the researchers of the present study. When comparing our results to those of previously published meta-analyses, only one significant association of actigraphy-estimated long sleep with an adverse outcome was observed after controlling the false-positive probability of multiple tests, while a number of diseases have been suggested to be associated with subjectively long sleep, even by meta-analyses [38]. In the UKB, we saw that nearly a quarter of the self-reported long sleepers actually slept no more than 6 h when estimated by objective measurement. Subjective recall of sleep traits especially sleep duration has long been suspected to bear systematic error [39]. The discrepancy between subjective and objective sleep durations may be attributed to the misunderstanding of the sleep concept. For example, some participants with difficulty falling asleep or keeping stable sleep may have spent long time in bed but have short real sleep. These objectively short sleepers, who bear a real increased disease risk, may misclassify themselves as subjectively long sleepers and introduce contamination to the effect estimation of long sleep. As evidenced by our analyses, this dramatic misclassification of sleep duration has introduced substantial bias to the estimation of effect size for a number of diseases, including stroke, ischemic heart diseases, cardiovascular disease, and depressive episode and recurrent depressive disorder. Moreover, several recent Mendelian randomization studies support this conclusion, as their results showed no causal effect of long sleep on cardiovascular diseases or stroke [40–42]. This finding may help resolve the uncertainty about the health effect of long sleep duration, which has been a concern of several clinical recommendations [43]. It may also underscore the necessity of viewing multiple sleep traits as a combination, because they may have inherent correlation [44], especially when common morbidity affecting different sleep traits exist, such as “insomnia with objectively short sleep duration”. As Tubbs et al. [45] demonstrated, there is an interaction between short sleep duration and insomnia severity on the risk of depression.

As a complex physiological process that occupies about one-third of human life, emerging evidence is indicating that different dimensions of sleep may have different effects in relation to different systems [11,12]. However, as illustrated in Fig. 2D, the existing literature has disproportionately focused on sleep duration rather than other sleep traits. Our analysis found sleep timing and sleep rhythm to correlate with a comparable or even greater number of diseases. More importantly, the associations of diseases with different sleep dimensions are highly independent, as 57.6% of diseases were associated with only one sleep dimension, suggesting that a skewed focus on sleep traits may lead to overlooking of some sleep-related diseases. Indeed, among the newly discovered associations in our study, most involved sleep rhythm—3 times as many as involved sleep duration. Recently, rhythmic oscillation has been proposed as one of the hallmarks of health [46]. A robust rhythm needs both a steady phase of oscillation and a robust amplitude of oscillation. In our study, it means sleeping with stable sleep timing every day (high interdaily stability) and sleeping at motionless night (high relative amplitude, when compared with vigorous daytime). As the circadian clock has been found to exist in almost all organs and to regulate physiological processes including but not limited to DNA repair, cell cycle, mitochondrial dynamics, metabolism, and specific functioning in certain organs or cells [47,48], it is no wonder that accumulating evidence suggests disruption of sleep rhythm to induce adverse effects on multiple physiological systems [38]. Sleep onset timing also deserves attention, as delaying sleep may increase exposure to artificial lighting and caloric intake at night, which may confer damage to human health [49]. Our results may suggest that the various dimensions of sleep traits deserve more attention in future studies and that pursuing comprehensive control of sleep traits may have greater benefit for health over merely ensuring adequate sleep duration. Fortunately, 4 of the 6 sleep traits are behavior related, which may be directly modified, and the remaining 2 can be improved when the first set is corrected [50], suggesting that sleep intervention may bring substantial health benefit. For example, the disruption of sleep rhythm can be substantially improved if the unhealthy habit of social networking media use before sleep (so-called Twitter jetlag) can be corrected [51]. Indeed, the estimated contribution of sleep traits to some common diseases is generally comparable to those of well-known risk factors, such as dementia (16.1% by sleep duration, 20.9% by obesity [52], and 7.2% to 15.9% by low education level [53]), cardiovascular disease (13.76% by relative amplitude [to ischemic heart diseases], 10.2% by low education level [54], and 6.7% to 10.7% by smoking [55]), Parkinson’s disease (31.68% by relative amplitude, 17% to 23% by pesticide/herbicide exposure, and 10% by repeated blows to the head for male [56]), and type 2 diabetes (22.39% by interdaily stability, 30% for Western dietary pattern [57], and 9% to 14% by smoking [58]).

In our study, sleep fragmentation (sleep efficiency and waking numbers) was found to be associated with a relatively small number of diseases. This may suggest that representing sleep fragmentation status is not an advantage of the current accelerometer technique. Since there has been no direct and entirely accurate way to comprehensively measure sleep–wake physiology in free-living humans, both subjective and objective sleep measurements are imperfect in some aspects. Integrative measurements taking advantage of each method may be a choice.

In our study, COPD was identified to associate with sleep rhythm and to have a substantial PAF, which was replicated in the independent NHANES population. Although one of the most important diseases worldwide [59], COPD has not previously been reported to correlate with sleep rhythm. However, some biological evidence may have existed. We found 10 respiratory diseases to show association with sleep, over half of which refer to inflammation and infection or related complications. On the other hand, several reviews have summarized the role of sleep in immune system function, the disruption of which may induce infectious disease and proinflammatory responses [43,59]. Our results also suggested that leukocytes, eosinophils, and C-creative protein have mediatory roles in the sleep–COPD relationship [60]. Furthermore, some cases of COPD are comorbid with asthma, meaning mechanisms by which sleep affects asthma, such as blood eosinophils, fraction of exhaled NO, and lung parenchymal abnormalities, may also contribute to COPD [40,41,60].

Mediation by inflammation could also help to partially explain the newly discovered relationship between sleep rhythm and acute kidney failure. Moreover, we also found that sleep rhythm may affect chronic kidney disease, diabetes, and primary hypertension, which are important risk factors of acute kidney failure and may consequently induce its occurrence [42,61]. Disrupted sleep rhythm may also suppress the rhythmic activity of xenobiotic metabolism in the liver [62], which may induce chemical-related kidney damage. It is worth noting that the 4 newly identified sleep-rhythm-related diseases such as COPD have caused substantial global burden in the world [63–66]. The most unfavorable quartile of sleep rhythm was associated with generally 1.5- to 2-fold disease risk (Fig. 4), with estimated PAFs by sleep rhythm to be over 15% (Table S6). These results further emphasize the well-known fact that an appropriate sleep pattern may comprehensively benefit the physiology of the human body, and sleep behavior can be a valuable intervention target. As to other trait–disease associations that have not ever been reported, their confirmation by independent studies may spark interest in figuring out the underlying mechanisms.

There are several major limitations in our study. First, the UKB study was not designed to be a representative of the nationwide population; only 5.45% of the invited participants attended the baseline, who were mainly middle-aged or elder and more susceptible to certain diseases [67], which may limit the generalizability of the results. Second, the accelerometry study took place in 2013 to 2015, while other covariates were measured in 2006 to 2010. However, a previous study reported that the covariates, except for medications, changed little over time [68]. Third, accelerometer measurements were taken only at a single time point, ignoring within-person changes or variability in movement behaviors over time. However, previous analyses of UKB data showed that sleep and physical activity patterns remained relatively stable over time. Fourth, there are possibilities of residual confounding. We calculated E-values to indicate to readers to interpret the associations with caution. Fifth, reverse causation bias cannot be ruled out, although sensitivity analyses were performed by excluding diseases developed within the first 2 years. We also replicated the newly identified disease associations with sleep rhythm in the independent NHANES population, which had a different age structure and BMI. While this may extend the generalizability of our findings, the NHANES study was also observational, and reverse causation bias may exist. Sixth, the literature search in our study included many cross-sectional studies, for which it was difficult to discern the temporal relationship between sleep traits and disease occurrence as many diseases have been found to disrupt sleep. This could have led to discrepancy when comparing our longitudinal results with the literature. Seventh, we only obtained external data to validate a subset of the newly identified trait–disease associations. The rest await replication in further studies. Finally, when identifying associations between sleep traits and individual diseases, the FDR method was used to control for the probability of type I error, but this method was not the most restrict. It may lead to false-positive mistakes in our analysis with objective sleep traits or weaken our capability to identify false-positive associations in the publications of subjective studies.

## Conclusion

The present study illustrates a landscape of 172 diseases associated with objectively measured sleep traits in adults. The results revealed that not only sleep duration but also other sleep traits contributed to a number of disease types with substantial attributable disease burden, emphasizing that comprehensive control of multiple sleep traits is necessary and may yield considerable health benefit. The objective sleep measurements can be a useful complement to sleep–disease studies, and integration of subjective and objective methods may provide more complete and accurate information about sleep’s health impact.

## Ethical Approval

The UKB study has approval from the North West Multi-Center Research Ethics Committee. All participants provided informed consent for the study to have their records linked to hospital admissions, cancer registries, and death registries.

## Data Availability

Details of how to access UK Biobank data and details of the data release schedule are available from https://www.ukbiobank.ac.uk.
