# Supplementary material for: Phenome-wide Analysis of Diseases in Relation to Objectively Measured Sleep Traits and Comparison with Subjective Sleep Traits in 88,461 Adults
Source: Health Data Sci. 2025 Jun 3;5:0161. doi: 10.34133/hds.0161 (PMC12131323; doi:10.34133/hds.0161)
Supplement: Supplementary 1 — Supplementary Methods Figs. S1 to S7 Tables S1 to S16 [file hds.0161.f1.zip › Figure S3.pdf]

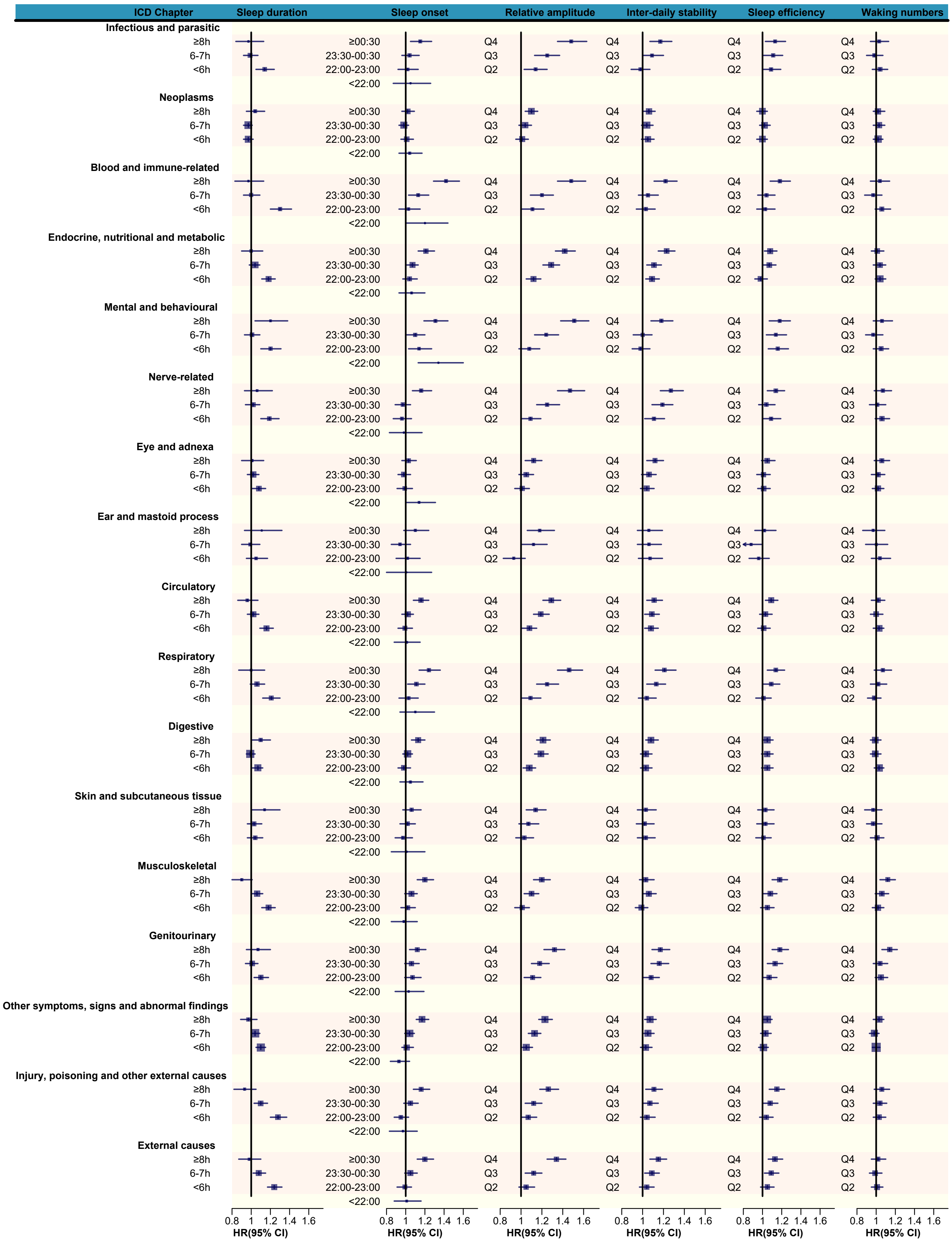
ICD Chapter
Sleep duration
Sleep onset
Relative amplitude
Inter-daily stability
Sleep efficiency
Waking numbers
Infectious and parasitic
Neoplasms
Blood and immune-related
Endocrine, nutritional and metabolic
Mental and behavioural
Nerve-related
Eye and adnexa
Ear and mastoid process
Circulatory
Respiratory
Digestive
Skin and subcutaneous tissue
Musculoskeletal
Genitourinary
Other symptoms, signs and abnormal findings
Injury, poisoning and other external causes
External causes
≥8h
6-7h
<6h
≥00:30
23:30-00:30
22:00-23:00
<22:00
Q4
Q3
Q2
0.8
1
1.2
1.4
1.6
HR(95% CI)
