# Supplementary material for: Phenome-wide Analysis of Diseases in Relation to Objectively Measured Sleep Traits and Comparison with Subjective Sleep Traits in 88,461 Adults
Source: Health Data Sci. 2025 Jun 3;5:0161. doi: 10.34133/hds.0161 (PMC12131323; doi:10.34133/hds.0161)
Supplement: Supplementary 1 — Supplementary Methods Figs. S1 to S7 Tables S1 to S16 [file hds.0161.f1.zip › Figure S6.pdf]

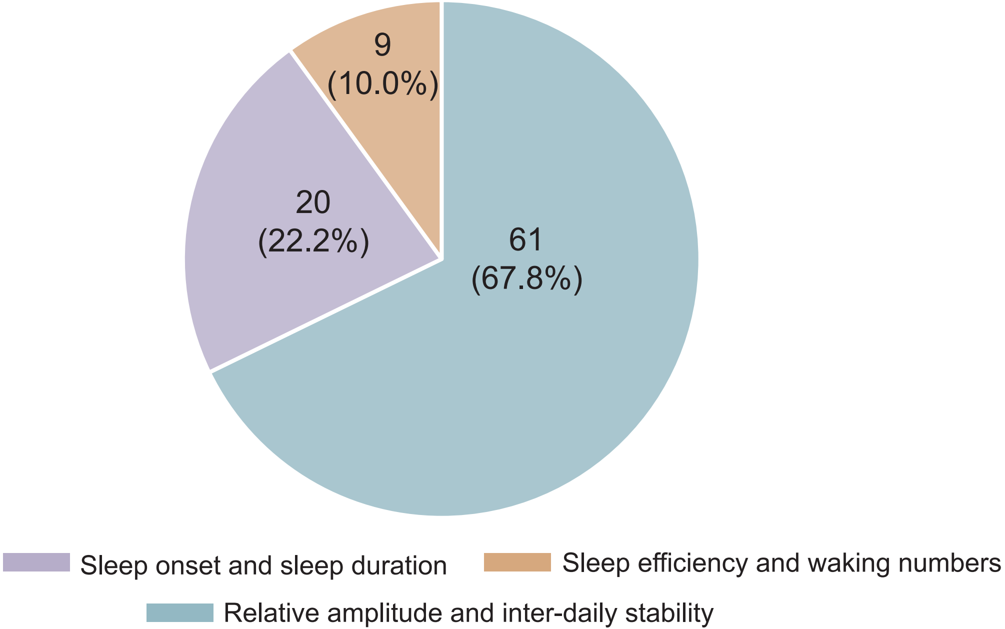
9
(10.0%)
20
(22.2%)
61
(67.8%)
Sleep onset and sleep duration
Sleep efficiency and waking numbers
Relative amplitude and inter-daily stability
