# Supplementary material for: Phenome-wide Analysis of Diseases in Relation to Objectively Measured Sleep Traits and Comparison with Subjective Sleep Traits in 88,461 Adults
Source: Health Data Sci. 2025 Jun 3;5:0161. doi: 10.34133/hds.0161 (PMC12131323; doi:10.34133/hds.0161)
Supplement: Supplementary 1 — Supplementary Methods Figs. S1 to S7 Tables S1 to S16 [file hds.0161.f1.zip › Supplementary Figures Captions.docx]

**Fig. S1. Inclusion and exclusion of study subjects from the UK Biobank dataset.**

**Fig. S2. Distribution and correlation of sleep traits.** (A). Distribution and correlation of sleep traits. Blue (left) and red (right) lines on the histograms denote the 5th and 95th percentiles. (B). Correlations of sleep traits using the Pearson method. Blue and red represent inverse and positive associations, respectively.

**Fig. S3. Associations between 17 candidate disease chapters and six sleep traits.** Cox proportional hazard models were applied to estimate hazard ratios (HR) and 95% confidence intervals (CI). The vertical line indicates the reference value of 1.

**Fig. S4. The 44 diseases with population attributable fraction (PAF) over 30% for a certain sleep trait.** PAF was calculated for each sleep trait on the associated diseases and sorted by magnitude.

**Fig. S5. Further analysis of the association of subjective long sleep with stroke and cardiovascular disease (stroke and ischemic heart disease), adjusted for objective sleep duration.** The subjective long sleepers were divided into four groups based on objective nocturnal sleep duration (<6 hours, 6-7 hours, 7-8 hours, and ≥8 hours), and separately compared to those who reported 7-8 hours subjective sleep duration.

**Fig. S6. Summary of the newly-discovered associations of diseases with sleep dimensions.**

**Fig. S7. Unweighted results for the associations of sleep rhythm with COPD and kidney failure in the NHANES study.**
