# Supplementary material for: Phenome-wide Analysis of Diseases in Relation to Objectively Measured Sleep Traits and Comparison with Subjective Sleep Traits in 88,461 Adults
Source: Health Data Sci. 2025 Jun 3;5:0161. doi: 10.34133/hds.0161 (PMC12131323; doi:10.34133/hds.0161)
Supplement: Supplementary 1 — Supplementary Methods Figs. S1 to S7 Tables S1 to S16 [file hds.0161.f1.zip › Supplementary Methods.docx]

**Accelerometer data and quality control**

Accelerometer data was drawn from a subsample, including 103,666 participants who accepted the invitation of wearing a wrist-worn AX3 triaxial accelerometer (Axivity, Newcastle upon Tyne, UK) for seven days during 2013-2015. Participants were required to wear the accelerometer on the wrist of their dominant hand. All participants provided informed consent for the study. The UK Biobank study has approval from the North West Multi-center Research Ethics Committee.

We excluded participants with unqualified accelerometry data, defining as: 1) being flagged by UK Biobank as having data problems (field 90002), poor wear time (field 90015), poor calibration (field 90016), or unable to calibrate activity data on the device worn itself requiring the use of other data (field 90017); 2) having number of data recording errors (field 90182), interrupted recording periods (field 90180), or duration of interrupted recoding periods (field 90181) was greater than the respective variable’s 3rd quartile + 1.5 × IQR; 3) having less than four valid weeknight (Sunday through Thursday) or less than one weekend night (Friday and Saturday); 4) phenotypes determined using the SPT-window (all phenotypes except RA and IS) had additional exclusions based on short (<3 h) and long (>12 h) mean sleep duration and too low (≤5) or too high (≥30) mean number of sleep episodes per night; leaving 88,461 participants for the main analysis. All measures were derived by processing raw accelerometer data in CWA format using GGIR (https://github.com/wadpac/GGIR/wiki/Publication-list)) (version 2.8.2) [16, 19]. In this study, three dimensions of sleep measures, including two sleep traits each, were derived: 1) measures of nocturnal sleep duration and sleep onset timing [16]; 2) measures of sleep rhythm (relative amplitude and inter-daily stability) [17]; 3) measures representative of sleep fragmentation, including sleep efficiency and nocturnal sleep episodes (waking number) [18].
