# Supplementary figures and images for: Phenome-wide Analysis of Diseases in Relation to Objectively Measured Sleep Traits and Comparison with Subjective Sleep Traits in 88,461 Adults
Source: Health Data Sci. 2025 Jun 3;5:0161. doi: 10.34133/hds.0161 (PMC12131323; doi:10.34133/hds.0161)

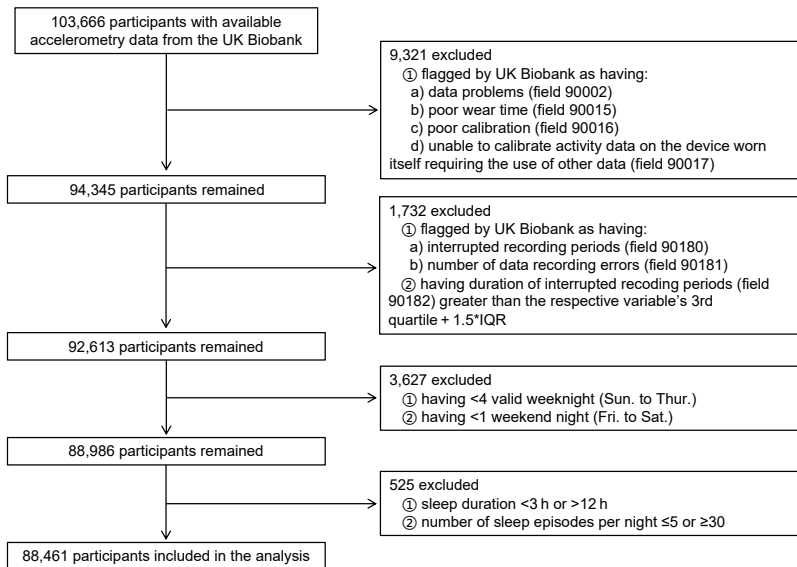

Supplement: Supplementary 1 — Supplementary Methods Figs. S1 to S7 Tables S1 to S16 [file hds.0161.f1.zip › Figure S1.pdf]

A

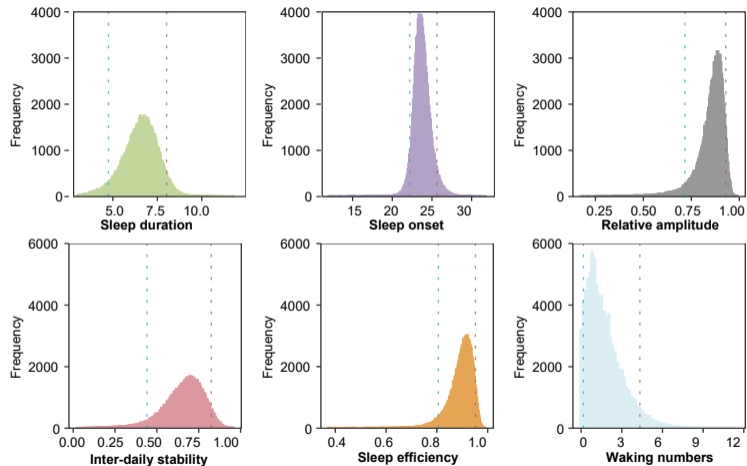

B

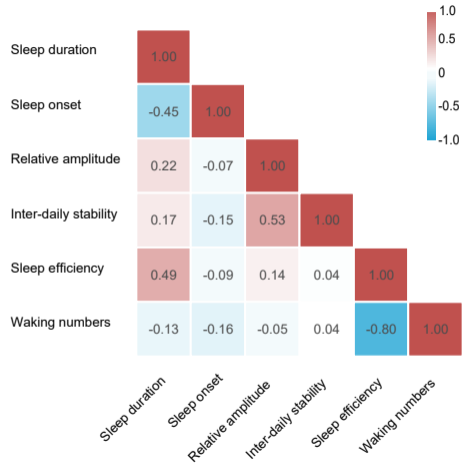

Supplement: Supplementary 1 — Supplementary Methods Figs. S1 to S7 Tables S1 to S16 [file hds.0161.f1.zip › Figure S2.pdf]

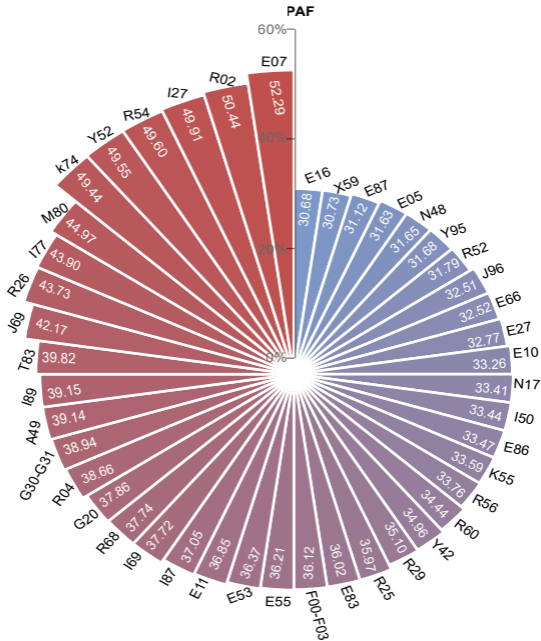

Supplement: Supplementary 1 — Supplementary Methods Figs. S1 to S7 Tables S1 to S16 [file hds.0161.f1.zip › Figure S4..pdf]

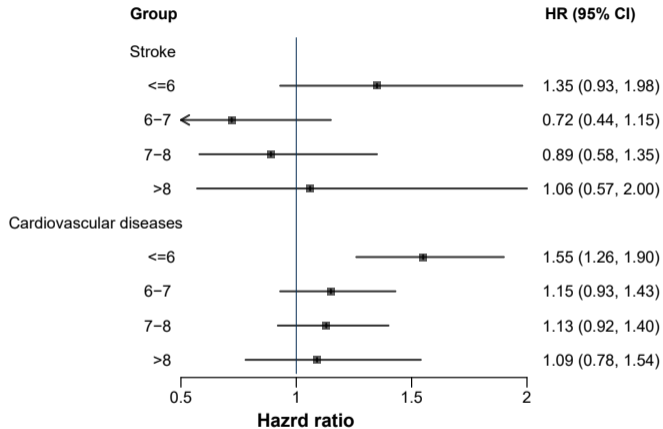

Supplement: Supplementary 1 — Supplementary Methods Figs. S1 to S7 Tables S1 to S16 [file hds.0161.f1.zip › Figure S5.pdf]

Relative amplitude

Inter-daily stability

COPD

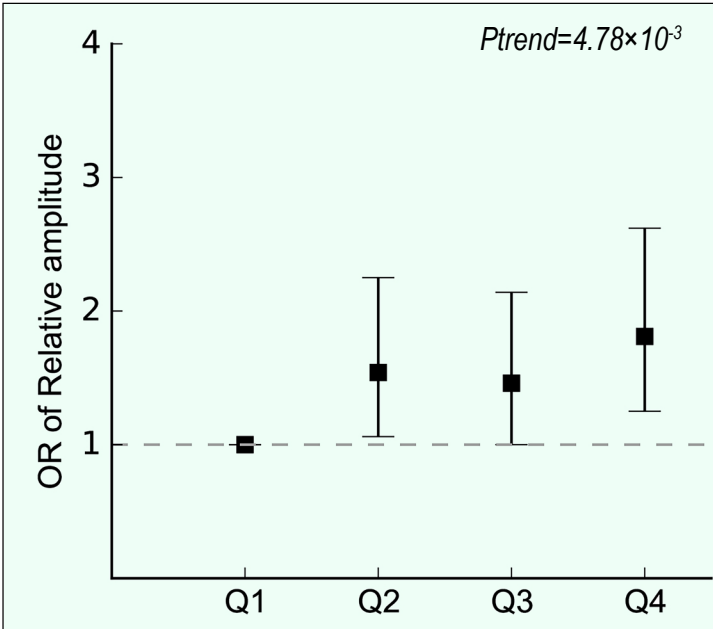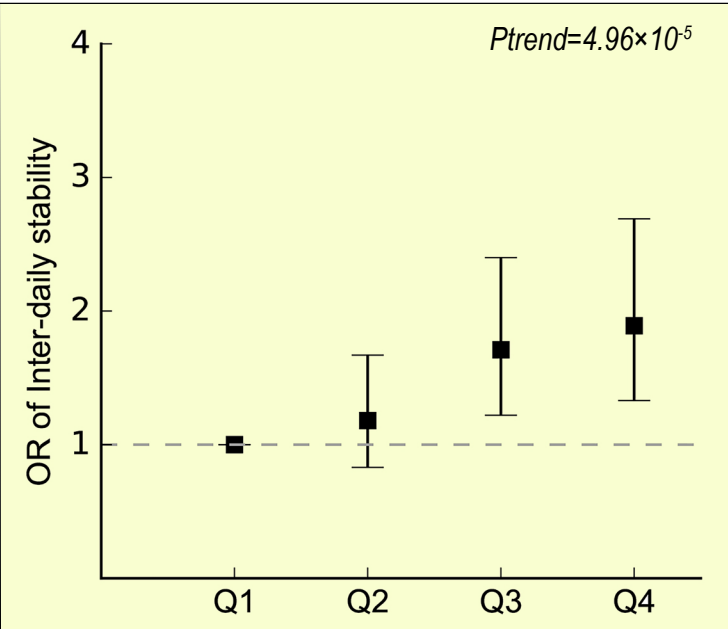

Kidney failure

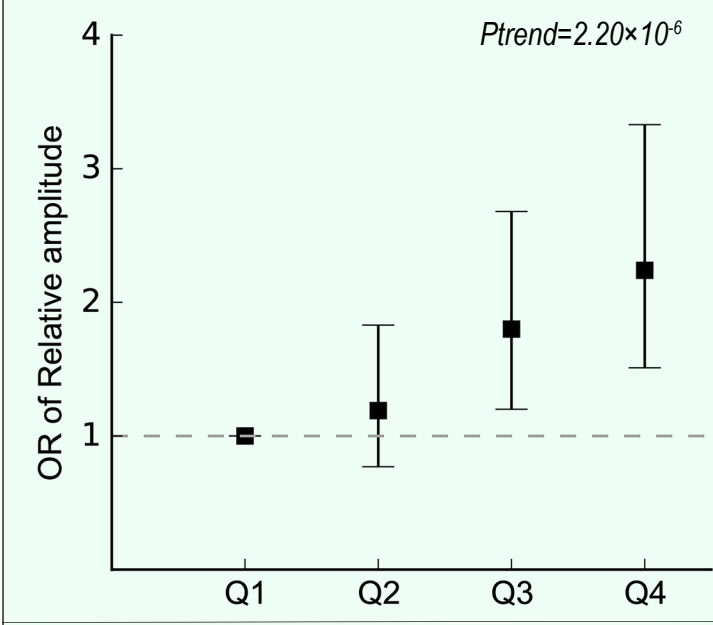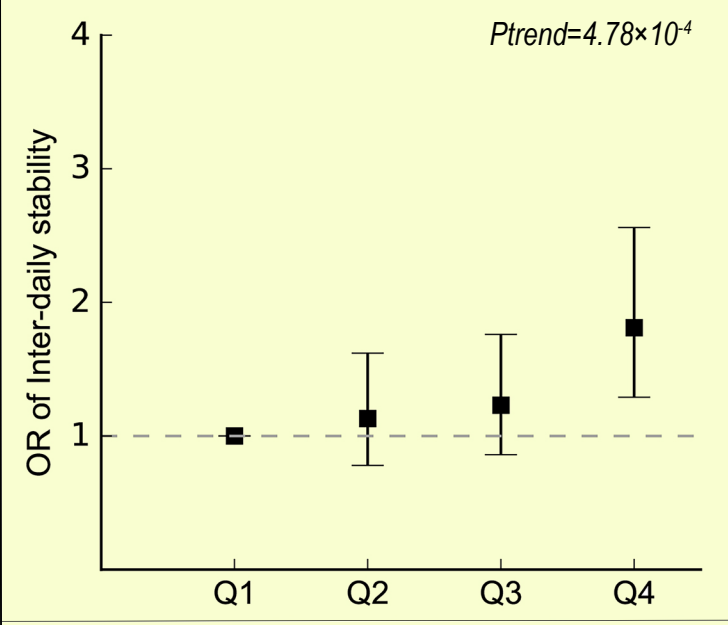

Diabetes

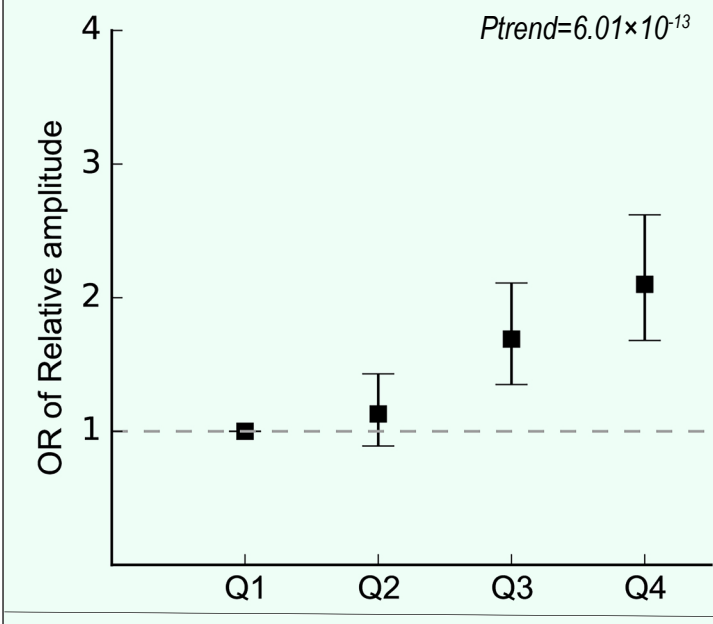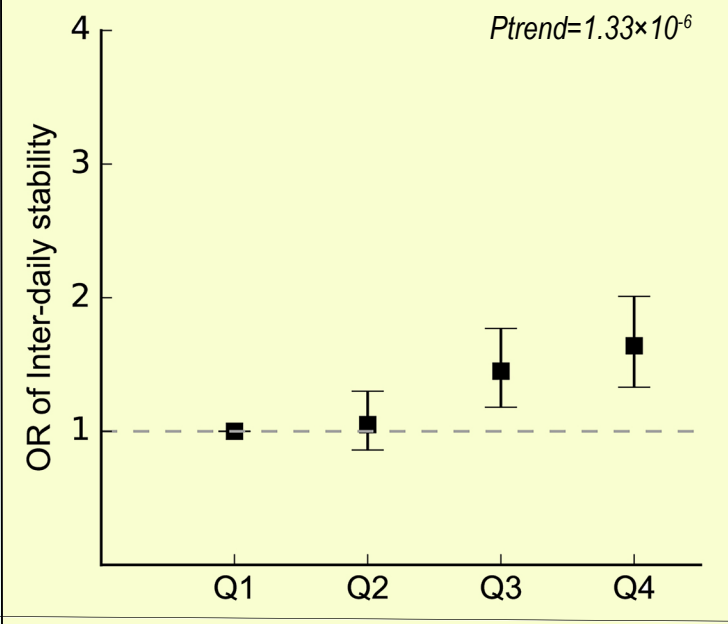

Depression

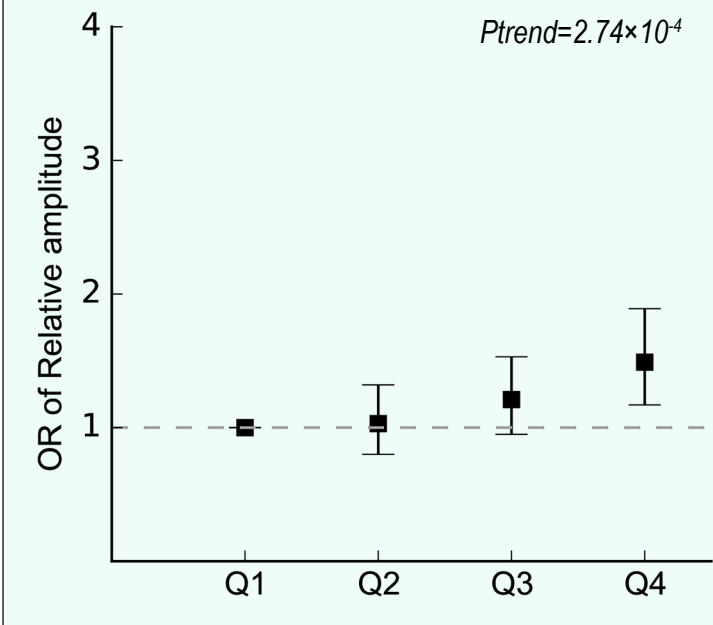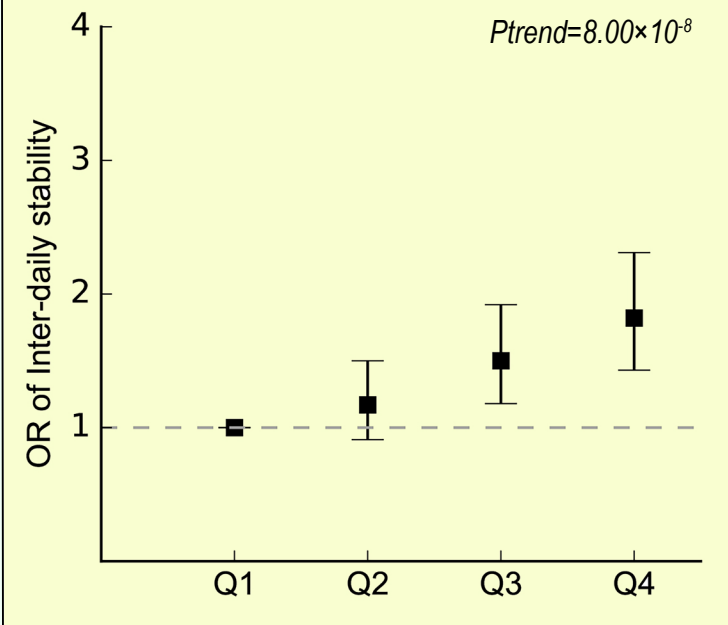

Supplement: Supplementary 1 — Supplementary Methods Figs. S1 to S7 Tables S1 to S16 [file hds.0161.f1.zip › Figure S7.pdf]
